# Mapping the different methods adopted for diagnostic imaging instruction at medical schools in Brazil

**DOI:** 10.1590/0100-3984.2015.0223

**Published:** 2017

**Authors:** Rubens Chojniak, Dominique Piacenti Carneiro, Gustavo Simonetto Peres Moterani, Ivone da Silva Duarte, Almir Galvão Vieira Bitencourt, Valdair Francisco Muglia, Giuseppe D'Ippolito

**Affiliations:** 1 PhD, Member of the Committee for Instruction, Continuing Education, and Residency of the Colégio Brasileiro de Radiologia e Diagnóstico por Imagem (CBR), Professor at the Faculdade de Medicina da Universidade Nove de Julho (Uninove), São Paulo, SP, Brazil.; 2 MD, Resident at Hospital Dante Pazzanese, São Paulo, SP, Brazil.; 3 MD, General Clinician, private practice, Santos, SP, Brazil.; 4 PhD, Professor at the Faculdade de Medicina da Universidade Nove de Julho (Uninove), São Paulo, SP, Brazil.; 5 PhD, Professor in the Graduate Program in Health Sciences at the A.C. Camargo Cancer Center, São Paulo, SP, Brazil.; 6 Tenured Professor, Member of the Committee for Instruction, Continuing Education, and Residency of the Colégio Brasileiro de Radiologia e Diagnóstico por Imagem (CBR), Associate Professor at the Faculdade de Medicina de Ribeirão Preto da Universidade de São Paulo (FMRP-USP), Ribeirão Preto, SP, Brazil.; 7 PhD, Member of the Committee for Instruction, Continuing Education, and Residency of the Colégio Brasileiro de Radiologia e Diagnóstico por Imagem (CBR), Adjunct Professor in the Department of Diagnostic Imaging at the Escola Paulista de Medicina da Universidade Federal de São Paulo (EPM-Unifesp), São Paulo, SP, Brazil.

**Keywords:** Schools, medical, Education, medical/standards, Program evaluation/methods, Diagnostic imaging

## Abstract

**Objective:**

To map the different methods for diagnostic imaging instruction at medical schools in Brazil.

**Materials and Methods:**

In this cross-sectional study, a questionnaire was sent to each of the coordinators of 178 Brazilian medical schools. The following characteristics were assessed: teaching model; total course hours; infrastructure; numbers of students and professionals involved; themes addressed; diagnostic imaging modalities covered; and education policies related to diagnostic imaging.

**Results:**

Of the 178 questionnaires sent, 45 (25.3%) were completed and returned. Of those 45 responses, 17 (37.8%) were from public medical schools, whereas 28 (62.2%) were from private medical schools. Among the 45 medical schools evaluated, the method of diagnostic imaging instruction was modular at 21 (46.7%), classic (independent discipline) at 13 (28.9%), hybrid (classical and modular) at 9 (20.0%), and none of the preceding at 3 (6.7%). Diagnostic imaging is part of the formal curriculum at 36 (80.0%) of the schools, an elective course at 3 (6.7%), and included within another modality at 6 (13.3%). Professors involved in diagnostic imaging teaching are radiologists at 43 (95.5%) of the institutions.

**Conclusion:**

The survey showed that medical courses in Brazil tend to offer diagnostic imaging instruction in courses that include other content and at different time points during the course. Radiologists are extensively involved in undergraduate medical education, regardless of the teaching methodology employed at the institution.

## Abstract (pt)

**Objetivo:**

Mapear os diferentes métodos para o ensino de diagnóstico por imagem nas escolas médicas brasileiras.

**Materiais e Métodos:**

Estudo transversal, realizado por meio de envio de questionários para os coordenadores de 178 escolas médicas brasileiras. As seguintes características foram questionadas: modelo de ensino; carga horária; infraestrutura; número de alunos e profissionais envolvidos; temas expostos; modalidades e políticas de ensino em diagnóstico por imagem.

**Resultados:**

Dos 178 questionários encaminhados, 45 (25,3%) foram respondidos, sendo 17 (37,8%) de escolas públicas e 28 (62,2%) de escolas privadas. O método de ensino de diagnóstico por imagem foi o modular em 21 (46,7%) escolas, o clássico (disciplina independente) em 13 (28,9%), o híbrido (clássico e modular) em 9 (20,0%) e outro método em 3 (6,7%). A disciplina diagnóstico por imagem é parte do currículo formal em 36 (80,0%) das escolas, curso eletivo em 3 (6,7%) e outra modalidade em 6 (13,3%). Os docentes envolvidos no ensino de diagnóstico por imagem são radiologistas em 43 (95,5%) das escolas.

**Conclusão:**

O levantamento mostrou que os cursos de medicina no Brasil tendem a oferecer o conteúdo de diagnóstico por imagem em unidades curriculares que associam outros conteúdos e em diferentes momentos do curso. Há um grande envolvimento de radiologistas no ensino de graduação, independentemente do método de ensino disponível na instituição.

## INTRODUCTION

Brazil, like other Latin American countries, has undergone an expansion of its higher education system, with an increase in the number of universities and the number of openings available. This expansion has also included medical education^(1)^. Since the beginning of the 2000s, the number of medical schools in Brazil has doubled, reaching more than 200, and the majority of them, approximately 60%, are private. Together, these schools graduate approximately 19,000 physicians per year^(1)^.

At the same time, the teaching of medicine has undergone substantial changes worldwide, mainly in the advancement of the curriculum structure, the diversification of teaching models, and the inclusion of technological tools in the list of teaching-learning strategies^(2-9)^. This scenario demands constant attention and organization of educational institutions to ensure the appropriate training of new physicians, avoiding excessive variation in the profile of professionals, as well as the perpetuation of teaching models, contents, and course hours that are not adapted to the new reality of the profession^(2,9,10)^.

An important consideration in medical education and that should be a reason for permanent analysis and reformulation in teaching models, is the incorporation of knowledge of new high complexity techniques in diagnostic and therapeutic practices, such as in the area of diagnostic imaging (DI), in a manner consistent with the evolution of the curriculum^(2,3,5,9-11)^. Among the many technological advances in the medical field in recent decades, the progress achieved in the DI field certainly figures prominently. Some of the greatest innovations in medicine occurred in this area, and this technological revolution accelerated the incorporation of DI techniques into clinical research strategies. In 2005, an estimated 60 million computed tomography scans were performed in the United States, a 20-fold increase over a period of 25 years. Nuclear medicine tests also tripled over the same period, reaching an estimated 20 million tests per year^(11-14)^.

DI resources are increasingly present in everyday medical practice, not only in clinical practice but also in scientific research. Currently, medical professionals often first come into contact with normal and pathological anatomy through imaging tests and many such tests also provide physiological and metabolic information that is essential for appropriate clinical decision-making^(3,11,12)^.

Despite the rapid adoption and popularization of imaging techniques in medical practice, the integration of DI into undergraduate medical courses has been uneven and slow in relation to technological advances, for various reasons, one of which is the divergence of opinions about the limit of complexity in subsidiary tests that should be taught at the undergraduate level^(2,5,11,15,16)^. Other authors have suggested that the ideal incorporation of the subject in undergraduate courses could be compromised by the difficulty in attracting radiologists to the academic activity, due to the rapid expansion of the specialty in the last decades, generating better job opportunities in the clinical area. That could also explain the inclusion of non-specialist professors in the teaching of this discipline^(3,5,12,17)^.

Although the importance of DI in undergraduate medical education is indisputable, the need for the formal requirement of a specific discipline of DI or the teaching of this content distributed in different curricular units throughout the medical course is widely discussed worldwide. The method of teaching DI is not even standardized at traditional centers such as those in Europe and the United States^(2,3)^. Studies have shown that there is growing interest in better integrating the discipline of DI into undergraduate medical curricula, and that there are many benefits of the early exposure of students to this area of knowledge, although only a few schools include instruction in this subject in a structured manner^(3,5,11,12,18)^.

Despite a lack of standardization, there has been a trend toward the formalization of DI in undergraduate courses in the United States and Europe, which aligns with the desire of students and professionals to become familiar with and monitor the technological evolution of imaging methods as a way of improving their professional activity, minimizing patient exposure to ionizing radiation, ensuring the safety of patients and healthcare professionals, and avoiding the costs associated with unnecessary tests^(2,16,19)^.

In Brazil, the Board of Higher Education of the Brazilian National Ministry of Education National Education Council is the organ that establishes the National Curricular Guidelines (NCGs) for undergraduate medical courses to be observed in the creation, development, and evaluation of the courses within the public and private higher education systems in the country^(20)^. The NCGs, instituted in 2001 and updated in 2014, set out the principles, foundations, and purposes of medical education, failing to establish mandatory minimum curricula, as was done previously, allowing institutions to offer content, without restrictions, in a way that is coherent with their potential^(20)^. Therefore, the way in which instruction is organized is a prerogative of each school, which can decide on the pedagogical model and the disciplines adopted in order to develop it. Schools can also determine the timing of the administration of subjects and disciplines, their respective schedules, and whether the corresponding courses will be required or elective. Consequently, we now have a situation in which there can be great diversity in the approach to medical education^(20)^.

Currently, various pedagogical models are applied in medical education and those models, in different ways, seek to follow the NCGs for the teaching of medicine^(2,4,5,10,21)^: the traditional model (conventional or classical), based on the classical medical disciplines; the problem-based learning (PBL) model; the modular model, based on systems and apparatus studies; the model that primarily uses simulation; and the hybrid model—the combination of the traditional model and one or more of the other models mentioned. This variation in pedagogical models adopted by different schools hinders perception of the specific content offered, the comparison of teaching results obtained, and the creation of support guidelines for the preparation of curricula^(2,5,11,12)^.

Article 5 of the current NCGs lists the skills and abilities that a physician requires in order to be able to practice medicine. Among the skills listed is the ability to perform diagnostic and therapeutic procedures based on scientific evidence, focusing on the optimization of clinical assessment, semiological resources, and contemporary therapies, requested hierarchically for comprehensive health care, which, in our view, underscores the importance of studies related to the teaching of diagnostic techniques in medicine^(20)^. In this context, there is a need for studies on teaching trends and outcomes for the different specific skills and for different teaching models. Studies conducted in various countries have analyzed the different approaches to DI instruction in undergraduate courses^(2,8,11,18,21)^. We have also identified some initiatives to standardize and update the specific content of such courses, which have stated the importance of training the professors involved and have cited their experience as a factor influencing education and the future choices made by medical students^(2,3,5,11,12,22,23)^.

In Brazil, there have been few studies addressing DI instruction at the undergraduate level. The analysis of this topic would benefit greatly from knowledge of the current state of teaching of this subject and its insertion into the academic curriculum of medical schools^(24)^. Given that, unfortunately, there is still no network of cooperation among medical schools to establish a discussion on teaching/learning methods in the area of DI; and the lack of national surveys, even with the increasing number of courses, and the fact that there already are guidelines and parameters in other countries for the different models of medical education, a nationwide work, checking the current reality, could facilitate the processes of planning, organization, and implementation of DI instruction in undergraduate courses at medical schools.

Therefore, the objective of this study was to map the different methods adopted for DI instruction at the undergraduate level in the medical schools of Brazil.

## MATERIALS AND METHODS

This was a cross-sectional observational study, approved by the Research Ethics Committee of the Universidade Nove de Julho. It was carried out under the auspices of the Committee for Instruction, Continuing Education, and Residency of the Colégio Brasileiro de Radiologia e Diagnóstico por Imagem (CBR) (Brazilian College of Radiology and Diagnostic Imaging).

A survey conducted on the e-MEC website of the Brazilian National Ministry of Education in September 2014 allowed the identification of 178 medical schools in Brazil and the e-mail addresses of their respective coordinators.

An invitation letter, an informed consent form, and a questionnaire were e-mailed to all of the medical school coordinators, via a service for the distribution, collection, and analysis of questionnaire data. If we received no response within three weeks, we made a second attempt by sending a new invitation.

The coordinators of the medical schools were informed in the consent form that the information collected in the questionnaire would be treated as confidential and that the individual data would not be disclosed or used to evaluate or compare institutions.

The questionnaire prepared was adapted based on previous studies on the subject, containing questions designed to collect the following data: date; description of institutions and their administration; teaching models; and the DI instruction method and its characteristics^(2,5,15)^.

Only the data obtained from questionnaires filled out by coordinators who agreed to participate in the study (i.e., those who gave written informed consent) were used for analysis.

The proposed questions sought to identify characteristics of DI instruction such as teaching model; total course hours; infrastructure; number of students and professionals involved; themes addressed; DI modalities covered; and education policies related to DI. For all categorical questions, the participant was allowed to select more than one alternative, and thus the total number of responses obtained for a given variable could be higher than the total number of questionnaires answered. We did not provide criteria for defining teaching models/methods or specialists, leaving the interviewees free to use their own criteria. In addition, for all questions, there was the possibility of including answers that were not contained in the list of options, as well as freeform comments.

Data were provided with descriptive statistical analysis.

## RESULTS

Of the 178 medical school coordinators who received questionnaires, 46 (25.8%) responded. Only one declined to participate. Therefore, we obtained data for 45 (25.3%) of the medical schools contacted. In all cases, those data were provided by the coordinators themselves.

Of the 45 schools evaluated, 28 (62.2%) were private and 17 (37.8%) were public, with the following geographical distribution: 19 (42.2%) were in the southeastern region of the country; 9 (20.0%) were in the southern region; 9 (20.0%) were in the northeastern region; 7 (15.6%) were in the central-west region; and 1 (2.2%) was in the northern region. The number of students admitted to each school per year ranged from 60 and 360.

Among the teaching methods adopted at each institution, the hybrid method was reported in 16 (35.6%), the traditional method was reported in 16 (35.6%), the PBL method was reported in 12 (26.7%), and other methods were reported in 6 (13.3%). Those other teaching methods were described as active methods in 3 (6.7%), in transition from the traditional to the PBL method in 2 (4.4%), and modular methods in 1 (2.2%). The respondents for five schools selected more than one response, all of which indicated PBL associated with other methods, 3 (6.7%) reporting the transition phase and 2 (4.4%) reporting active methods.

Regarding the DI teaching method, the modular method (teaching distributed throughout modules) was described in 21 (46.7%) of the responses, the classical method (as an independent discipline) was described in 13 (28.9%), the hybrid (classical plus modular) method was described in 9 (20.0%), and another teaching method was described in 3 (6.7%). These other methods were described as being integrated with PBL during the course at 2 (4.4%) of the schools and as being classical but inserted within another discipline (clinical medicine) at 1 (2.2%).

Instruction in DI was referred to as part of the formal curriculum in 36 (80.0%) of the responses, as an elective course in 3 (6.7%), and as another modality in 6 (13.3%). Other modalities were referred to as being integrated into other required classical or modular disciplines in 5 (11.1%) or as a required and elective subject in 1 (2.2%).

As for the timing of DI instruction in the course (in years and in the course stage), we found that the content was distributed in different modules in 24 (53.3%) of the courses; throughout the course in 9 (20.0%); together with basic subjects in 8 (17.8%); during internship in 4 (11.1%); and another distribution in 8 (17.8%). In eight questionnaires, two answers to this question were given, indicating other specific combinations for the timing of DI instruction.

Of the coordinators of the 45 institutions, 14 (31.1%) responded that they offer 0-50 hours of DI instruction in theoretical classes, compared with 50-100 hours reported by 17 (37.8%) and > 100 hours reported by 6 (13.3%). Eight of the coordinators reported difficulty in estimating the total course hours, because the practical DI content is inserted into several modules or discussions throughout the course in the PBL model. Not all schools offer DI instruction in practical classes. In 39 (88.7%) of the responses, there were indications of practical DI classes with course hours ranging from 0-50 hours in 15 schools (33.3%) to 50-100 hours in 9 (20.0 %) and > 100 hours in 10 (22.2%). Five of the coordinators reported difficulty in estimating the total course hours, because the practical content of DI is inserted into several modules or discussions throughout the course in the PBL model.

**Figure 1:**
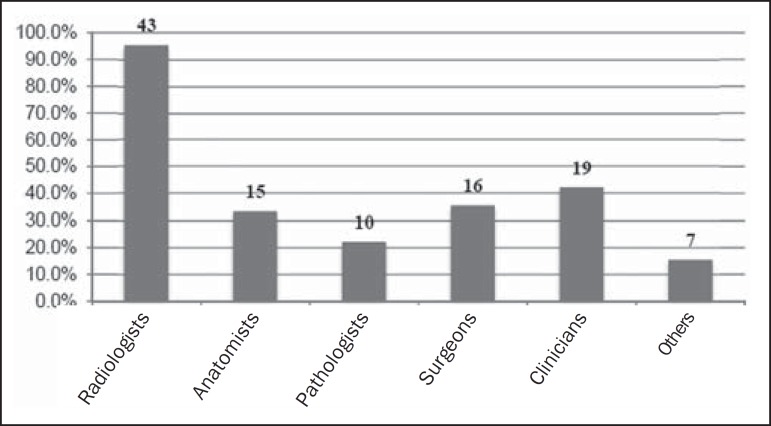
Professionals involved in DI teaching at the undergraduate level, in proportions and total number of responses.

Regarding professors, virtually all of the respondents indicated that they have radiologists involved in teaching DI at some point in the course. However, there is also a large number of other professionals who are in some way responsible for teaching DI content (Figure 1).

**Figure 2:**
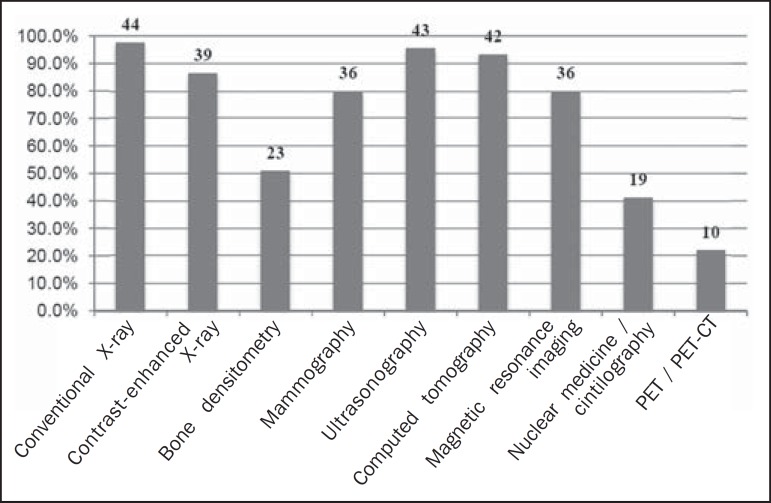
Modalities of DI methods available for teaching at medical schools, in proportions and total number of responses.

The diversity of teaching sites was thus presented in the responses: classroom, in 42 (93.3%); hospitals, in 23 (51.1%); in on-site laboratories, in 21 (46.7%); at clinics, in 16 (35.6%); in off-site laboratories, in 6 (13.3%); and in other settings, in 3 (6.7%). Other responses included primary health care clinics, in 3 (6.7%) and computer labs, in 1 (2.2%). The imaging modalities available for teaching are described in Figure 2.

When asked about the existence of interaction with other teaching materials, respondents indicated the following: books, in 40 (88.9%); seminars, in 26 (57.8%); discussion of scientific articles, in 25 (55.6%); lectures, in 24 (53.3%); follow-up of examinations or preparation of in-service reports, in 19 (42.2%); software, in 18 (40.0%); performing procedures under supervision, in 11 (24.4%); and conferences on the topic, in 7 (15.6%).

Scientific studies and thesis work in the field of DI were considered rare by 35 (77.8%) of the respondents, common by 10 (22.3%), and very common by none.

## DISCUSSION

This study attempted to instigate discussion on DI, a very important area in medical education, and to shed light on the characteristics of DI instruction in Brazil.

One of the first challenges was getting responses from medical school coordinators, and it was necessary to resend the invitations in some cases. At present, we do not have sufficient data to relate this negative finding to a lack of interest in discussing the subject or to internal policies of these institutions. However, it is relevant that only about a quarter (27.2%) of the institutions contacted returned the questionnaire sent.

The fact that most (62.2%) of the responding institutions were in the private sector reflects the proportion of private schools in Brazil, which is estimated at 60%^(1)^.

In Brazil, the number of openings in medical courses is regulated by the Brazilian National Ministry of Education, which explains the numerical discrepancy of students between one school and another. This factor may be related to differences in resources available for teaching in this area up to the fourth year, being compensated for during the internship (compulsory in-service internship) carried out in the last two years of the course.

Regarding the teaching method, the results show that there is no uniformity of pedagogical models in Brazil, which is consistent with the current legislation; that is, consistent with the NCGs for undergraduate medical courses, which allows the adoption of different models^(20)^.

The results also show that 33 courses provide DI instruction in association with other disciplines or subjects, and that only 13 offer a specific discipline, which is consistent with the adoption of new pedagogical models. However, when we associate these data with those for the professionals involved in DI instruction, we observe that, regardless of the DI teaching model, nearly 100% of the courses have professors that specialize in this area, and it can be inferred that even in the courses that have professors with other backgrounds, institutions still give primary responsibility for teaching DI to the radiologist, and that other professionals teach the application of imaging in their areas of activity or participate in multiprofessional curricular units. We can also conclude that there are Brazilian radiologists who are available and interested in working in undergraduate education. This result is similar to that of a study by the European Radiology Society, which reports the existence of radiologist professors in 98% of the evaluated courses^(2)^. The authors of that study stated that in the so-called modern curriculum (46% of courses) the student has already had contact with DI in the first year and has a gradual development of knowledge in this area toward the end of the course, whereas the so-called conventional curriculum (accounting for 59% of the total course work) does not offer DI content in a specific curricular unit, but rather as an optional discipline^(2)^. We found that approximately half of the medical schools evaluated started teaching DI in the first two years, either as a basic subject or in the PBL and active teaching methods. We also noted that most of the schools provide DI content at two or more time points during the course. This is in line with the findings of studies conducted in Europe, which have identified great variation in the timing of DI instruction in the curriculum, as well as the association between early and longitudinal exposure to DI content in so-called modern curriculum models^(2,5)^. In fact, in our sample, only about a third of schools indicated the adoption of the traditional teaching method.

The total course hours that medical schools report they devote to DI teaching was another item that showed great variation in our analysis and in that of other authors^(2,5,15)^.

Regarding teaching locations, results show that more than 90% of the respondent courses in this study offer DI activities in the classroom, complemented in a variety of ways, such as in laboratories, clinics, and hospitals.

In relation to the imaging modalities available for DI instruction at undergraduate level, it is noteworthy that not all institutions employed conventional or contrast-enhanced X-rays, as would be expected. Nevertheless, we can consider that more than 80% of the respondents provide the most used modalities in attending the most important Brazilian National Ministry of Health programs that involve strategies of screening and early diagnosis. This findings, in particular, should be carefully evaluated, since we can discuss whether or not an undergraduate medical student, considering the focus on general education and the costs of implementing advanced techniques, should or should not have contact with all available modalities and whether that would be fundamental in the formation of clinical reasoning skills.

One suggestion to reduce economic pressure and allow the student to come into contact with innovative modalities in imaging would be to adopt the use of simulators and computer labs, which can maximize student time for learning and allow the application of teaching strategies based on the simulation of selected cases, motivating students to be more interested in the area and perhaps collaborate to increase academic production in the area, which is much higher in developed countries than in Brazil^(11,12,21,24)^.

The interest of the students can be quantified by the number of academic associations created in the fields of radiology and DI, 32 such associations having been registered on the CBR website by the end of 2015^(25)^. The creation of academic associations is an alternative found by the students themselves to overcome the deficiency of DI teaching during the undergraduate course. The leagues are created and organized by students and oriented by professors and professionals in the area. The dissemination of DI in undergraduate courses, the opportunities to exercise practical activities, and the incentive to do research are the essence of the motivation for the intense participation of students in the academic associations.

This survey has some limitations to be considered. Only 27.2% of the medical schools in the country were evaluated. Therefore, the results obtained may not be representative, given that the schools that did not return the questionnaire may have opted not to do so because they do not have a well structured DI teaching, which would represent a selection bias. Because it was an initial mapping and we therefore did not want to make the questionnaire too extensive, we decided not to include questions regarding detailed data, such as the academic qualification of those involved in radiology instruction and the degree of student interest in the specialty, as assessed by their participation in monitoring programs, scientific initiation programs, and relevant academic associations.

The mapping of the current undergraduate DI instruction in Brazil, based on the responses obtained in the present study, allows us to draw some important conclusions. The medical courses in Brazil appear to adopt a great variety of methods and total course hours for DI instruction, with a tendency to adopt curricular units that associate the teaching of DI with other content and at different time points during the course. We also found that almost all courses have DI specialist professors working in this teaching area.

The teaching of DI, like all areas of medical knowledge, needs to be constantly updated, in view of the current and future NCGs for medical undergraduate courses, focused on the training of the general practitioner and on the programs sponsored by the Brazilian National Ministry of Health. Within its proposal to disseminate and support the teaching of topics related to the specialty, the CBR, a national entity which officially represents the specialty in Brazil, can create strategies to monitor the evolution of medical education in the country and equip institutions and specialists who work in medical teaching, thus helping spark the interest of students, increase academic production, and improve the quality of medical performance in DI in Brazil.
